# 1159. Pharmacokinetics, Safety, and Tolerability of Imipenem/Cilastatin/Relebactam in Pediatric Participants With Confirmed or Suspected Gram-negative Bacterial Infections: A Phase 1b, Open-label, Single-Dose Clinical Trial

**DOI:** 10.1093/ofid/ofab466.1352

**Published:** 2021-12-04

**Authors:** John S Bradley, Nataliia Makieieva, Camilla Tøndel, Emmanuel Roilides, Matthew S Kelly, Munjal Patel, Pavan Vaddady, Alok Maniar, Ying Zhang, Amanda Paschke, Joan R Butterton, Luke F Chen

**Affiliations:** 1 University of California San Diego, San Diego, California; 2 Kharkiv National Medical University, Kharkiv, Kharkivs’ka Oblast’, Ukraine; 3 Haukeland University Hospital, Bergen, Hordaland, Norway; 4 Aristotle University and Hippokration General Hospital, Thessaloniki, Thessaloniki, Greece; 5 Duke University Medical Center, Durham, North Carolina; 6 Merck & Co., Inc., Kenilworth, New Jersey

## Abstract

**Background:**

Imipenem/cilastatin/relebactam (IMI/REL) is approved for treating hospital-acquired/ventilator-associated bacterial pneumonia, complicated urinary tract infection, and complicated intra-abdominal infection in adults. This study assessed single-dose pharmacokinetics (PK), safety, and tolerability of IMI/REL in neonatal and pediatric participants with confirmed or suspected gram-negative bacterial infections.

**Methods:**

This was a phase 1, open-label, non-comparative study (NCT03230916). Age- and weight-adjusted dosing is summarized in Table 1. The primary objective was to characterize the PK profiles for imipenem and relebactam after a single intravenous dose of IMI/REL. PK parameters were analyzed using population modeling. The PK target for imipenem was the percent time of the dosing interval that the unbound plasma concentration exceeded the minimum inhibitory concentration (%fT >MIC) of ≥30% (MIC used, 2 µg/mL). The PK target for relebactam was an area under the curve (AUC)/MIC ratio >8 (MIC used, 2 µg/mL), corresponding to AUC0-24h >58.88 μM∙h. Safety and tolerability were assessed for up to 14 days after drug infusion.

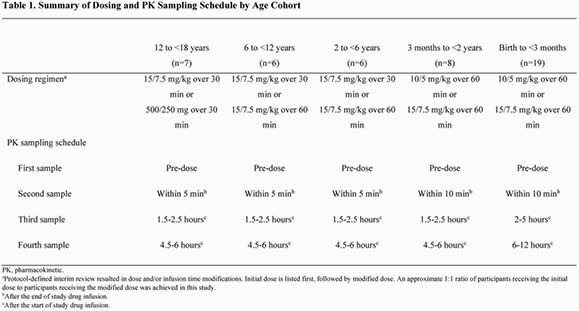

**Results:**

Of the 46 participants who received IMI/REL, 42 were included in the PK analysis. The mean plasma concentration-time profiles for imipenem and relebactam were generally comparable across age cohorts (Figure). For imipenem, the geometric mean %ƒT >MIC ranged from 50% to 94% and the mean maximum concentration (C_max_) ranged from 65 μM to 126 μM (Table 2). For relebactam, the geometric C_max_ ranged from 33 μM to 87 μM and mean AUC_0-6h_ ranged from 51 μM·h to 159 μM·h across the age cohorts (Table 2). IMI/REL was well tolerated with 8 (17.4%) participants experiencing ≥1 adverse events (AE) and 2 (4.3%) participants experiencing AE that were deemed drug related by the investigator. Drug-related AE were increased alanine aminotransferase, increased aspartate aminotransferase, anemia, and diarrhea, which were non-serious, mild in severity, and resolved within the follow-up period of 14 days.

Figure 1

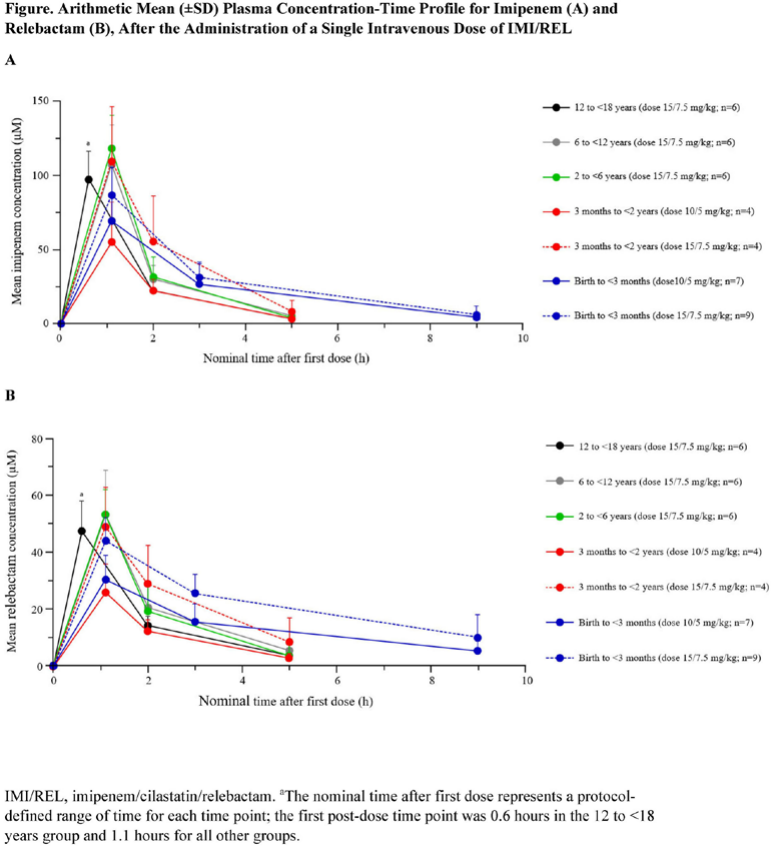

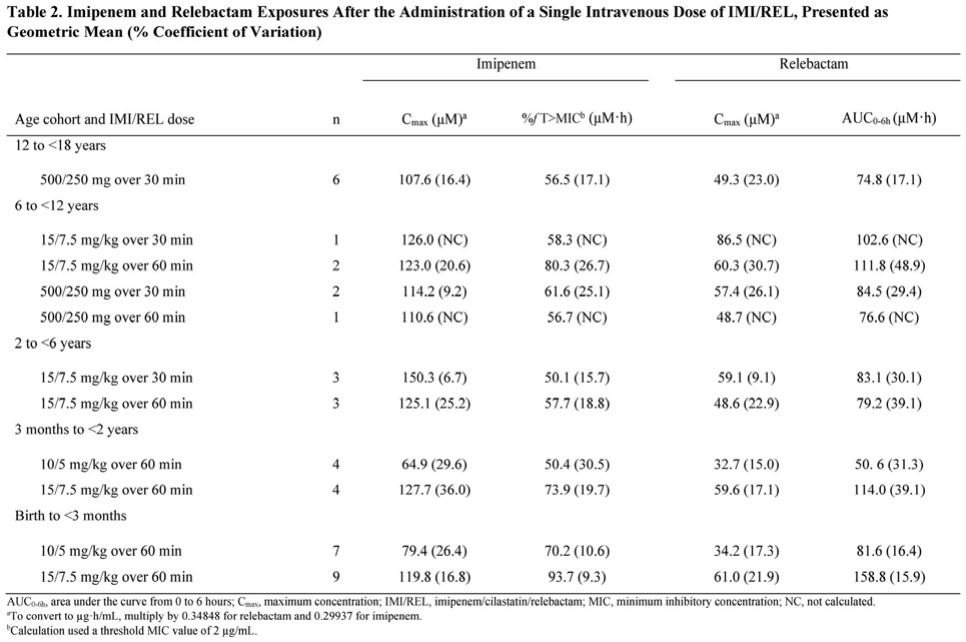

**Conclusion:**

Imipenem and relebactam exceeded the pediatric plasma PK targets across pediatric age cohorts in the study; the single doses of IMI/REL were well tolerated. These results will inform IMI/REL dose selection for further pediatric clinical evaluation.

**Disclosures:**

**Camilla Tøndel, MD, PhD**, **Merck & Co., Inc.,** (Grant/Research Support) **Emmanuel Roilides, MD, PhD, FIDSA, FAAM, FESCMID, FECMM, FISAC**, **Merck Sharp & Dohme Corp.** (Consultant, Grant/Research Support) **Matthew S. Kelly, MD, MPH**, **Merck Sharp & Dohme Corp.** (Consultant, Grant/Research Support) **Munjal Patel, PhD**, **Merck Sharp & Dohme Corp.** (Employee, Shareholder) **Pavan Vaddady, PhD**, **Merck Sharp & Dohme Corp.** (Employee) **Alok Maniar, MD, MPH**, **Merck Sharp & Dohme Corp.** (Employee, Shareholder) **Ying Zhang, PhD**, **Merck & Co., Inc.** (Employee, Shareholder) **Amanda Paschke, MD MSCE**, **Merck Sharp & Dohme Corp.** (Employee, Shareholder) **Joan R. Butterton, MD**, **Merck Sharp & Dohme Corp.** (Employee, Shareholder) **Luke F. Chen, MBBS MPH MBA FRACP FSHEA FIDSA**, **Merck** (Employee)

